# Supplementary material for: Optoelectronic Trajectory Reconfiguration and Directed Self‐Assembly of Self‐Propelling Electrically Powered Active Particles
Source: Adv Sci (Weinh). 2023 Apr 17;10(16):2206183. doi: 10.1002/advs.202206183 (PMC10238198; doi:10.1002/advs.202206183)
Supplement: Supplementary file 1 — Supporting Information [file ADVS-10-2206183-s006.pdf]

ADVANCED SCIENCE
Open Access

Supporting Information

for *Adv. Sci.*, DOI 10.1002/advs.202206183

Optoelectronic Trajectory Reconfiguration and Directed Self-Assembly of Self-Propelling Electrically Powered Active Particles

*Sankha Shuvra Das and Gilad Yossifon**

Supporting Information

Optoelectronic Reconfiguration and Directed Self-Assembly of Self-Propelling Electrically-Powered Active Particles

Sankha Shuvra Das[1] and Gilad Yossifon[1]*

[1]School of Mechanical Engineering, Tel-Aviv University, Tel-Aviv 69978, Israel

* Corresponding author: gyossifon@tauex.tau.ac.il

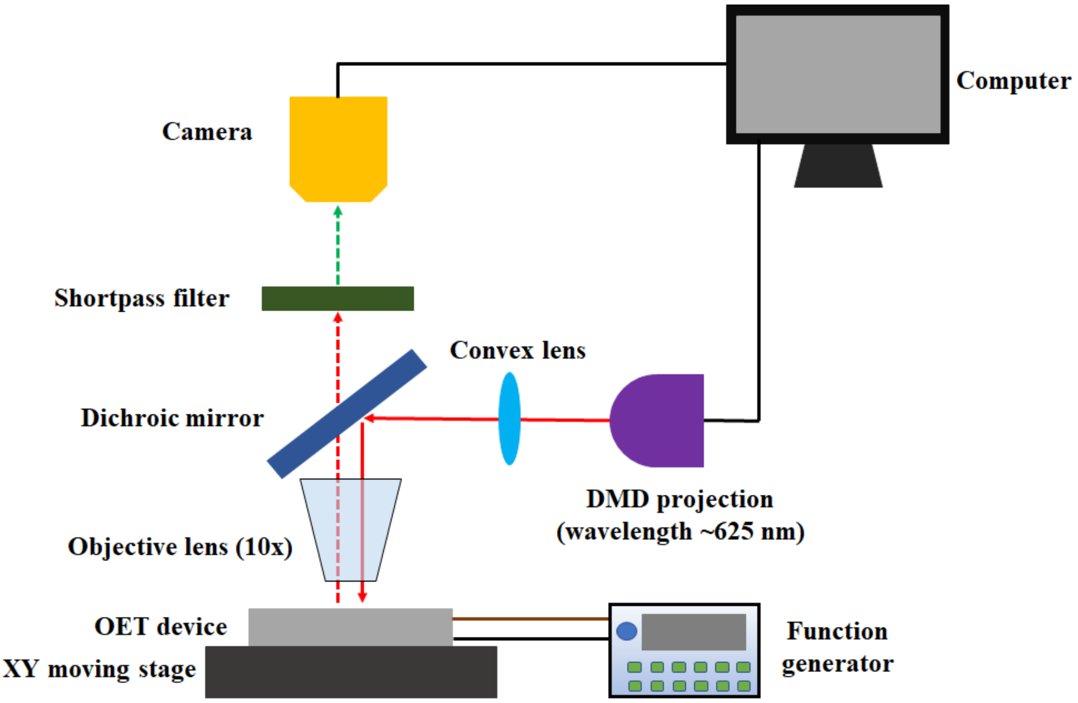

Fig. S1: Schematic of the OET experimental setup

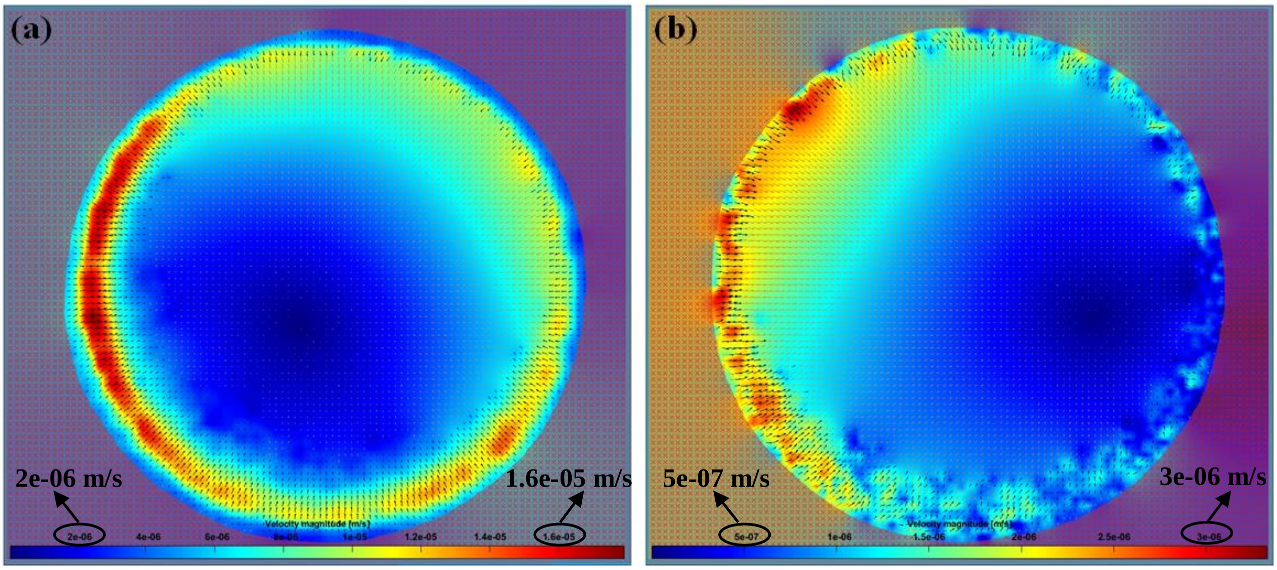

Fig. S2: Microscale particle image velocimetry (µPIV) characterization of the electrohydrodynamic generated flow field. The mean velocity magnitude (color plot) and velocity vector field of 1µm polystyrene tracer particles as measured through µPIV analysis of 100 frames under an applied voltage of 10Vpp and frequency of (a) 10 kHz or (b) 100 kHz.

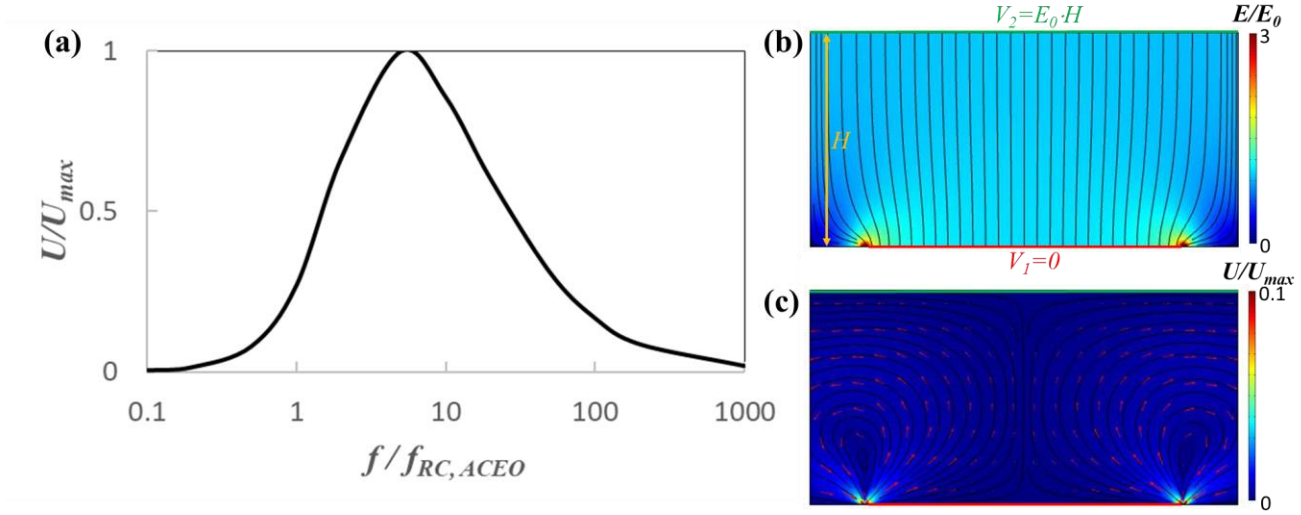

Fig. S3: Numerical simulations of alternating current electro-osmotic (ACEO) flow at the optically patterned electrode. (a) Frequency dispersion of the ACEO-induced velocity versus the frequency (normalized by the RC frequency of the ACEO). Numerical simulations depicting the surface and streamline plot of the normalized (b) electric and (c) velocity fields obtained for $f/f_{RC,ACEO}$=157 (i.e. f=10 kHz).

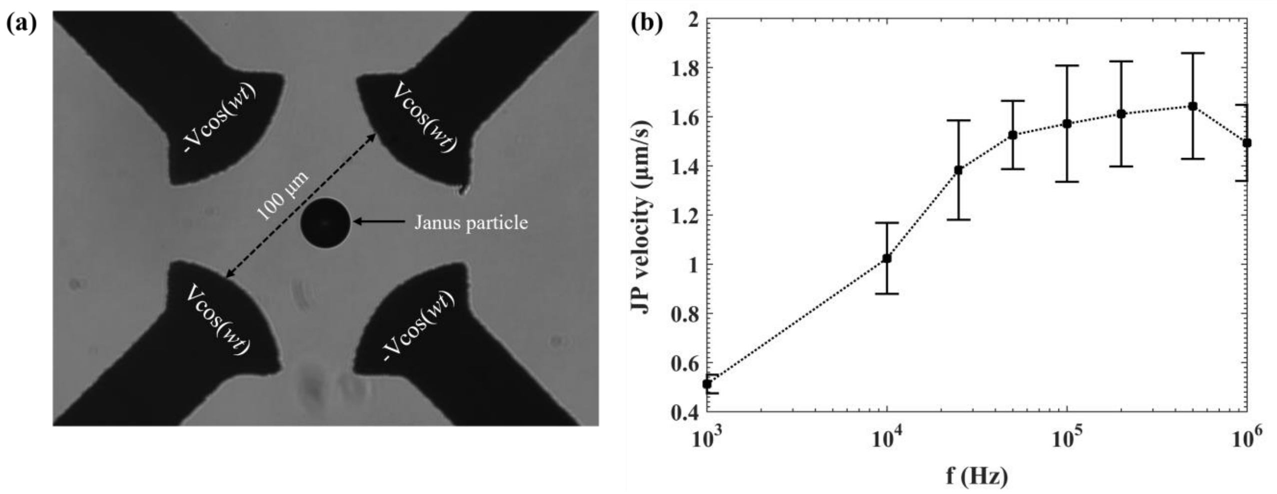

Fig. S4: Characterization of Janus particle (27 µm) dielectrophoretic (DEP) behavior. (a) Microscopic image of the quadrupolar electrode array setup used to investigate the DEP response of a JP. (b) DEP velocity of the JP determined from the average translation velocity within the interrogation region 10-20 µm from the quadrupolar electrode array edge. The applied electrical voltage was 0.5Vpp and frequency 1 kHz-1MHz. The plot shows the positive DEP (pDEP) response of the JP within the entire examined frequency range. A low-conduction electrolyte of 50 µM KCl with 0.1% (v/v) Tween-20 was used.

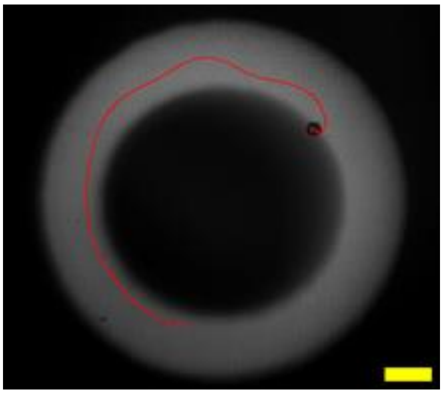

Fig. S5: JP trajectory within a ring-shaped optical pattern under ICEP motion. An applied field of 10Vpp, 10 kHz. Scale bar: 100 µm.

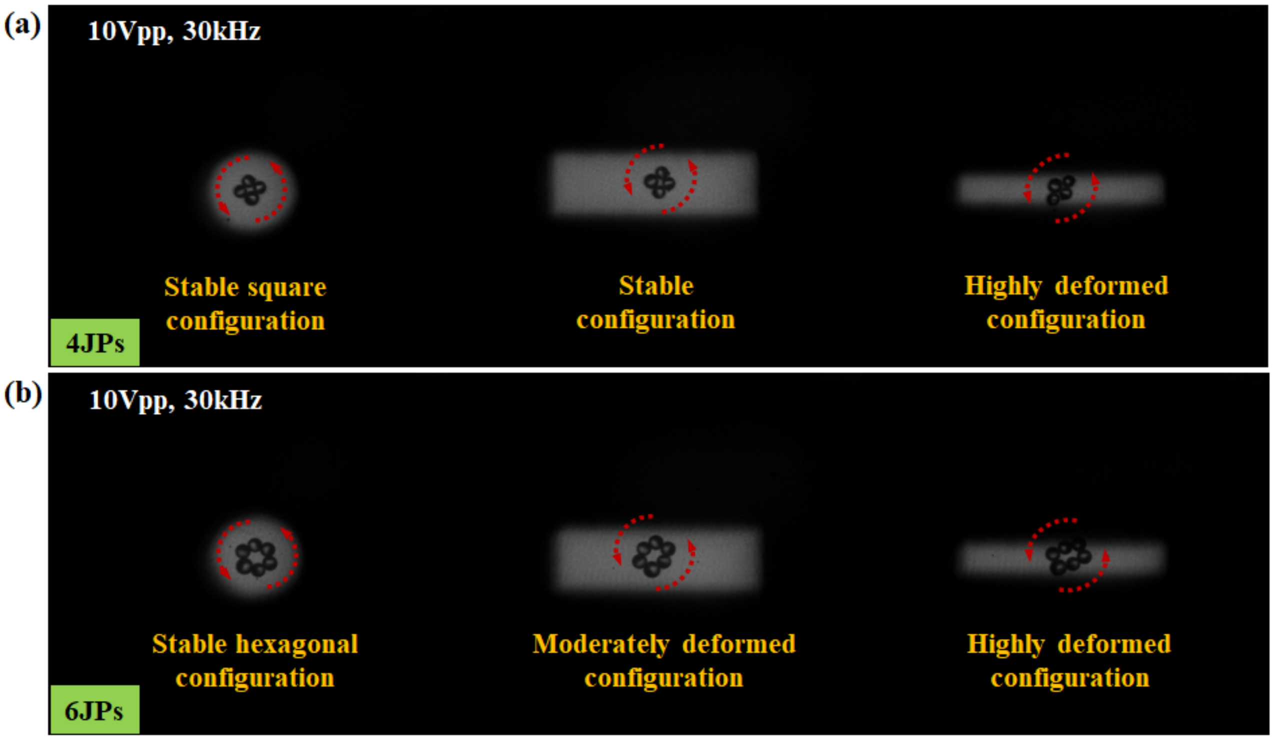

Fig. S6: The effect of confinement on the assembled active structure. Effect of confinement achieved by reducing the size of the optically patterned region to that of the assembled structure comprised of (a) 4 JPs or (b) 6 JPs, resulting in its deformation relative to the non-constrained case.

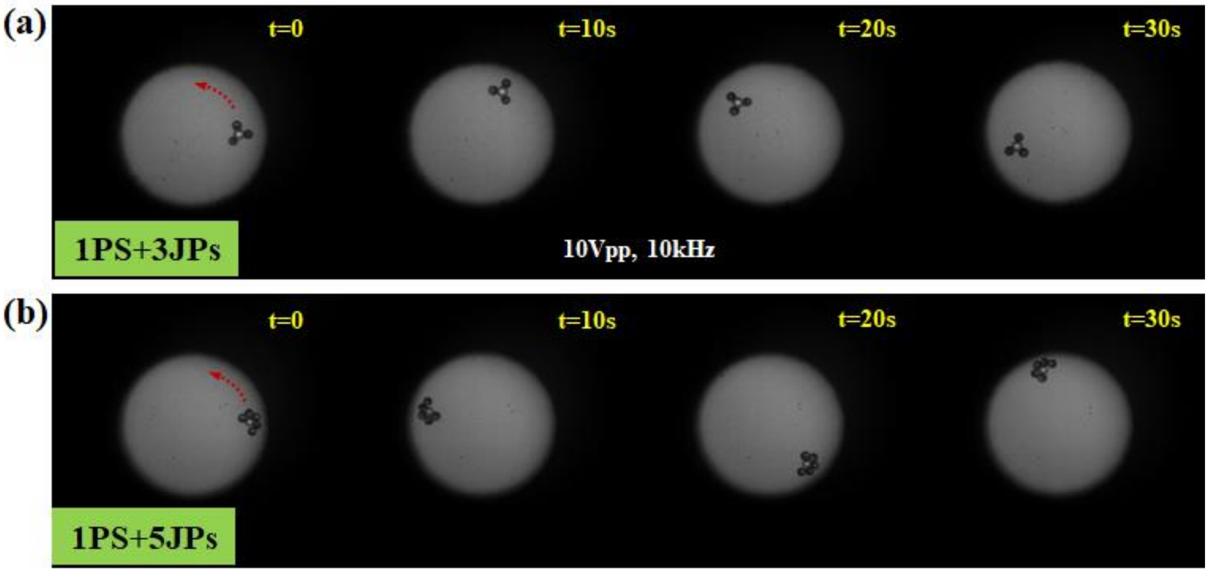

Fig. S7: Assembled hybrid structure combined of active JPs with a passive particle. The shape of the assembled hybrid structure comprised of (a) 3 or (b) 5 active 27 μm in diameter JPs and a passive polystyrene particle and its self-propelling motion within an optically defined region.

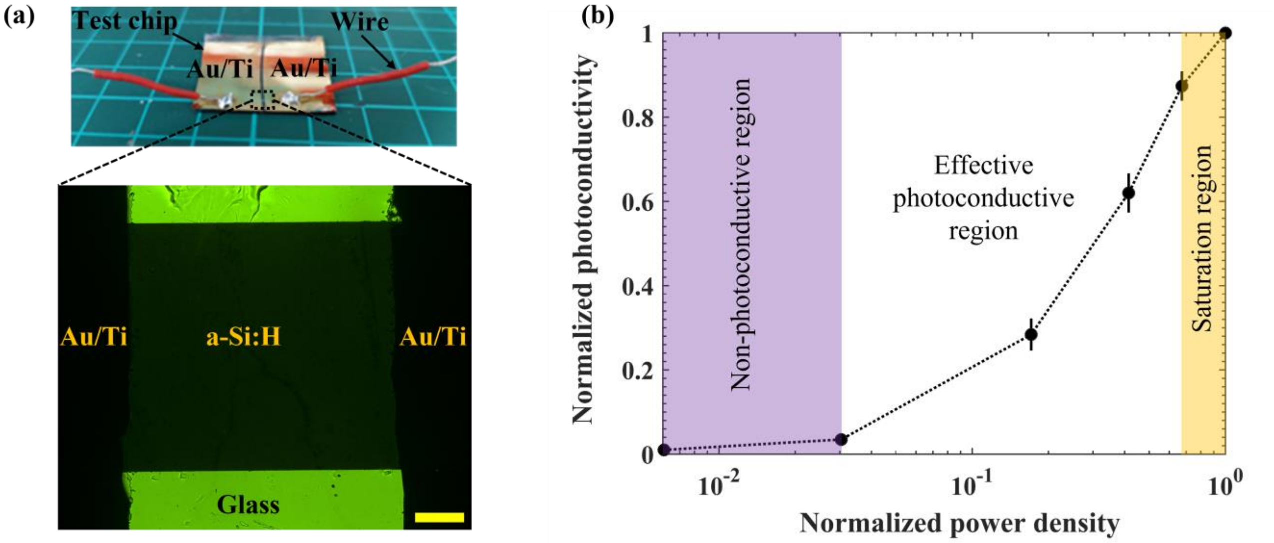

Figure S8: Measurement of the dependency of the photoconductive substrate's conductivity on the light intensity. (a) To test the photoconductivity of a-Si:H (deposited on a bare glass slide), first, a predefined structure of a-Si:H layer is fabricated using reactive ion etching (RIE) followed by electrode deposition (comprising of 50nm Ti and 200nm Au layers) onto it. Inset shows the microscopic image of the a-Si:H test structure (area: ~536 × 502 μm^2) and the deposited Au/Ti electrodes; scale bar: 100μm. (b) The measured normalized photoconductivity of the a-Si:H test structure versus the normalized power density of the illuminated red light (via an external light source with red filter). Photocurrents were measured for a constant applied voltage of 5V using Keithley 2636A source meter.

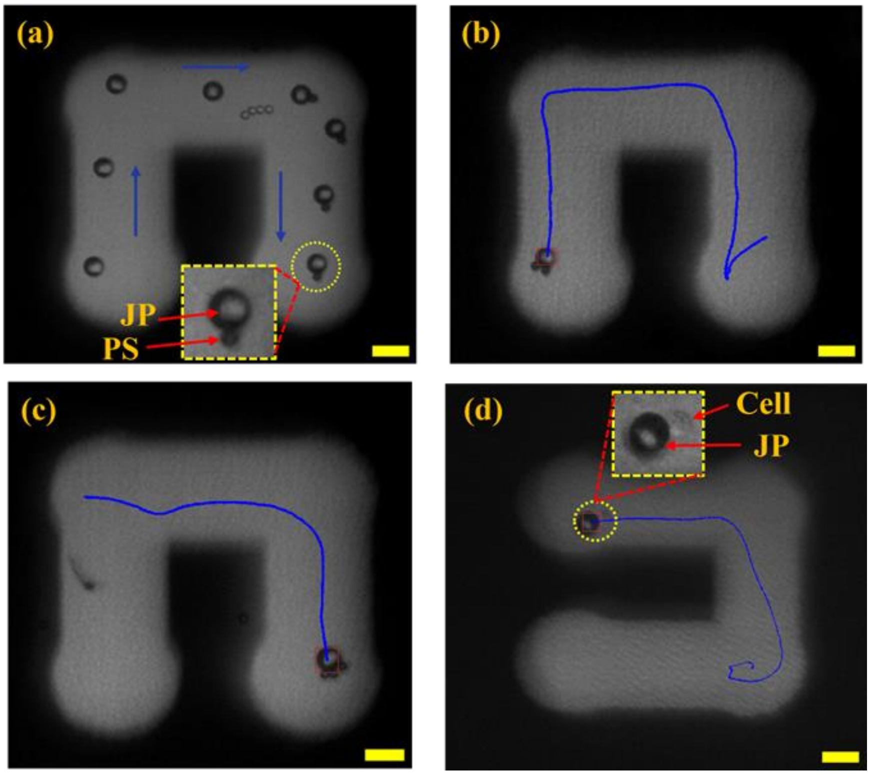

Figure S9: Demonstration of cargo (synthetic and biological) loading, transportation and delivery using a JP propelling within an optoelectronically controlled trajectory. (a) Superimposed sequential microscopic images of a single cargo (10µm polystyrene particle (PS)) loading at the equator of the JP's metallic hemisphere (via negative dielectrophoresis) followed by transportation and delivery of the cargo to other location (inset shows the magnified image of the polystyrene particle loaded onto the JP). Multiple cargo manipulation using the optoelectroniclly controlled JP for multiple cargos: (b) 2 and (c) 3 PS particles. See also supplementary video S7. (d) Optoelectronically controlled JP based manipulation of a fixed 293T cancer cell trapped at the equator of the JP's dielectric hemisphere via positive dielectrophoresis followed by transportation and delivery from one location to other (inset shows a magnified image of the cell loaded onto the JP). See also supplementary video S8. All the experiments were conducted in a low-conductivity electrolyte of 50 µM KCl with 0.1% Tween-20 with an applied electric field of 10Vpp, 100kHz. Scale bar: 50µm.

Supplementary Videos:

Supplementary Video S1: JP motion characteristics inside an illuminated floating electrode under low (10kHz) and high (100kHz) AC field frequency (corresponding to Figure 2a). The applied electric potential was 10Vpp.

Supplementary Video S2: JP motion characteristics inside a circular beam of varying sizes (corresponding to Figure 3a). The applied AC electric field was 10Vpp, 10kHz and 100kHz.

Supplementary Video S3: JP motion characteristics in optically patterned geometric channels (circular/square/hexagonal shaped) (corresponding to Figure 4a). The applied AC electric field was 10Vpp, 100kHz.

Supplementary Video S4: JP motion control in multiple optically patterned wells by sequentially linking them with each other (corresponding to Figure 4b).

Supplementary Video S5: Control of the interaction between multiple JPs by sequentially linking optically patterned wells (corresponding to Figure 4c).

Supplementary Video S6: Demonstration of JP self-assembly (corresponding to Figure 5a). The applied electric field was 10Vpp, 10kHz.

Supplementary Video S7: Demonstration of an optically controlled trajectory of a JP that acts as a cargo carrier of polystyrene particles (PS) of 10μm (corresponding to Figure S9a-c). The applied electric field was 10Vpp, 100kHz.

Supplementary Video S8: Demonstration of an optically controlled trajectory of a JP that acts as a cargo carrier of fixed 293T cancer cell (corresponding to Figure S9d). The applied electric field was 10Vpp, 100kHz.
